# <em>Leucaena leucocephala</em> fermented with <em>Lactiplantibacillus plantarum</em> TISTR1284 as a sustainable fishmeal substitute enhances immune responses and resistance to <em>Aeromonas hydrophila</em>infection in giant freshwater prawns (<em>Macrobrachium rosenbergii</em>)

**DOI:** 10.14202/vetworld.2026.2876-2898

**Published:** 2026-07-10

**Authors:** Arnon Pudgerd, Laorrat Phuapittayalert, Kamonwan Jongsomchai, Sirilak Sanpa, Sirikarn Sanpa, Paiboon Panase, Pornpan Pumirat, Sukanya Saedan, Rapeepun Vanichviriyakit, Charoonroj Chotwiwatthanakun

**Affiliations:** 1 Division of Anatomy, School of Medical Sciences, University of Phayao, Maeka, Muang, Phayao 56000, Thailand.; 2 Unit of Excellence in Plant and Aquaculture, University of Phayao, Maeka, Muang, Phayao 56000, Thailand.; 3 Division of Microbiology, School of Medical Sciences, University of Phayao, Maeka, Muang, Phayao 56000, Thailand.; 4 Division of Fisheries, School of Agriculture and Natural Resources, University of Phayao, Phayao 56000, Thailand.; 5 Unit of Excellence “Physiology for the Sustainable Production and Management of Aquatic Animals” Division of Fisheries, School of Agriculture and Natural Resources, University of Phayao, Phayao 56000, Thailand.; 6 Department of Microbiology and Immunology, Faculty of Tropical Medicine, Mahidol University, Bangkok 10400, Thailand.; 7 Department of Anatomy, Faculty of Science, Mahidol University, Rama VI Road, Bangkok 10400, Thailand.; 8 Center of Excellence for Shrimp Molecular Biology and Biotechnology (Centex Shrimp), Faculty of Science, Mahidol University, Rama VI Road, Bangkok 10400, Thailand.; 9 Nakhonsawan Campus, Mahidol University, Nakhonsawan 60130, Thailand.

**Keywords:** Aeromonas hydrophila, disease resistance, fishmeal replacement, giant freshwater prawn, immune response, Lactiplantibacillus plantarum, Leucaena leucocephala, sustainable aquaculture

## Abstract

**Background and Aim:**

The increasing cost and limited availability of fishmeal have intensified the search for sustainable alternative protein sources in aquaculture. Fermentation of plant-based feed ingredients can improve their nutritional value and functional properties. This study evaluated the effects of dietary inclusion of *Leucaena leucocephala* leaves fermented with *Lactiplantibacillus plantarum* TISTR1284 as a partial fishmeal substitute on immune responses, intestinal health, and resistance to *Aeromonas hydrophila* infection in giant freshwater prawns (*Macrobrachium rosenbergii*).

**Materials and Methods:**

Adult *M. rosenbergii* were randomly assigned to three dietary treatments: a control diet and diets in which 10% or 20% of fishmeal was replaced with fermented *L. leucocephala*. Prawns were fed the experimental diets for 2 weeks before challenge with *A. hydrophila*. Total hemocyte counts, phenoloxidase activity, hematopoietic tissue proliferation, immune-related gene expression, intestinal histology, antioxidant properties of the diets, and post-challenge survival were evaluated.

**Results:**

Fermentation enhanced the nutritional quality of *L. leucocephala* by increasing essential amino acid content and reducing mimosine concentration. Diets containing fermented *L. leucocephala* exhibited greater antioxidant activity, total phenolic content, and total flavonoid content than the control diet. Prawns receiving fermented diets showed significantly increased total hemocyte counts, enhanced hematopoietic cell proliferation, elevated crustacean hematopoietic factor expression, and increased phenoloxidase activity. The expression of key immune-related genes, including *IMD*, *Relish*, *HSP70*, *Cu/Zn-SOD*, and anti-lipopolysaccharide factor (*ALF*), was upregulated, whereas *TRAF6* and *Dorsal* expression was moderated during infection. Intestinal morphology remained normal, indicating that dietary inclusion of fermented *L. leucocephala* did not adversely affect gut structure. Following *A. hydrophila* challenge, prawns fed fermented diets exhibited a 26.14% reduction in mortality compared with the control group, demonstrating improved disease resistance.

**Conclusion:**

Partial replacement of fishmeal with *L. leucocephala* leaves fermented by *L. plantarum* TISTR1284 enhanced innate immune responses, improved antioxidant status, and increased resistance to *A. hydrophila* infection in *M. rosenbergii* without compromising intestinal health. Fermented *L. leucocephala* represents a promising sustainable functional feed ingredient for freshwater prawn aquaculture and may contribute to reducing dependence on fishmeal in commercial production systems.

## INTRODUCTION

Protein is an essential nutrient in aquaculture diets for crustaceans because it directly influences growth performance, immune function, and cellular processes such as tissue repair, renewal, and reproduction [1]. In *Macrobrachium rosenbergii*, the recommended dietary crude protein level for achieving optimal growth is approximately 35% of the total diet [2]. Fishmeal remains the predominant protein source in commercial aquafeeds because of its balanced amino acid profile, high digestibility, and low content of anti-nutritional factors (ANFs) [3, 4]. However, increasing aquaculture production and the growing demand for fish-derived products have contributed to rising fishmeal costs and concerns regarding the environmental and ecological consequences of overfishing [5, 6]. Consequently, rising price volatility and limited availability of fishmeal have intensified the search for sustainable, cost-effective alternative protein sources for aquaculture feeds [7, 8]. Plant-derived protein ingredients, including soybean, canola, blue-green microalgae, and cottonseed, have been extensively investigated as potential alternatives to fishmeal in sustainable aquaculture systems because of their favorable protein content and availability [3, 4, 9].

*Leucaena leucocephala*, a leguminous plant belonging to the family Fabaceae, is widely distributed throughout tropical and subtropical regions of Asia, Africa, and the Americas [10]. The plant contains relatively high protein concentrations (25.25%–30.81%) and provides essential amino acids, including lysine, leucine, proline, and serine, making it a potentially valuable feed ingredient [10]. In marine shrimp (*Penaeus vannamei*), dietary inclusion of 5%–20% *L. leucocephala* leaf powder did not adversely affect animal health, biochemical composition, or meat quality [9]. Similarly, replacement of fish protein with 25%–50% *L. leucocephala* leaf meal devoid of mimosine did not negatively affect the health of Nile tilapia [11]. Furthermore, fish fed diets containing 33% *L. leucocephala* seed powder exhibited enhanced immune responses during infection with *Vibrio harveyi* and *Pseudomonas aeruginosa* [12]. Beyond its nutritional value, *L. leucocephala* contains bioactive phytochemicals, particularly flavonoids, which possess antioxidant and immunomodulatory properties [13]. Flavonoids have been reported to induce erythropoietin gene expression and exhibit strong anti-inflammatory and antioxidant activities [14]. In aquatic animals, these compounds can interact with hemocyte receptors and activate intracellular signaling pathways associated with immune defense mechanisms [15]. Collectively, previous studies have demonstrated that partial replacement of fishmeal with up to 20% *L. leucocephala* leaf meal can be implemented without causing severe adverse effects on animal health or growth performance [9].

Despite these advantages, the application of plant-derived proteins in aquafeeds remains constrained by the presence of ANFs, including phytates, trypsin inhibitors, lectins, and other compounds that may reduce palatability, impair nutrient utilization, and adversely affect growth and immune performance [16]. Fermentation has emerged as an effective strategy for improving the nutritional quality and functionality of plant materials through biochemical transformation and detoxification [17]. Among the microorganisms used for this purpose, lactic acid bacteria (LAB), including species of *Lactiplantibacillus*, *Aerococcus*, and *Streptococcus*, have received considerable attention because of their safety and ability to enhance nutrient bioavailability [17, 18]. Fermented products are generally recognized as safe, nutritionally enriched, and stable during storage. Controlled fermentation using selected LAB strains has therefore been proposed as a valuable approach for producing high-quality functional feed ingredients [17].

*Lactiplantibacillus plantarum* is a particularly promising microorganism because of its capacity to biotransform phenolic-rich substrates and generate bioactive compounds with potent antioxidant activity. This bacterium facilitates the conversion of hydroxybenzoic acids, hydrolyzes tannins to gallic acid, and promotes the transformation of hydroxycinnamic acids into biologically active metabolites [18]. In addition to its well-recognized probiotic properties in humans, *L. plantarum* has demonstrated beneficial effects in aquaculture by enhancing growth performance, improving immune responses, and increasing disease resistance through the production of antimicrobial compounds that suppress pathogenic bacteria [19]. In *M. rosenbergii*, dietary supplementation with *L. plantarum* has been shown to improve host-associated microbiota, feed utilization, carcass composition, immune status, and survival following infection with *Aeromonas hydrophila* [19].

Although the nutritional and immunological benefits of *L. leucocephala* and *L. plantarum* have been independently documented, limited information is available regarding the combined application of these two components as a fermented functional feed ingredient in crustacean aquaculture. Previous studies have primarily focused on direct inclusion of *L. leucocephala* leaf meal or probiotic supplementation alone, whereas the effects of *L. leucocephala* fermented with *L. plantarum* on immune modulation, antioxidant status, intestinal health, and disease resistance have not been investigated in *M. rosenbergii*. Furthermore, the influence of fermented *L. leucocephala* on hematopoietic activity, phenoloxidase-mediated defense responses, and immune-related gene expression during bacterial infection remains poorly understood. Addressing these knowledge gaps is essential for evaluating the feasibility of fermented plant-based proteins as sustainable alternatives to fishmeal in freshwater prawn production systems.

Therefore, this study was conducted to evaluate the effects of partial replacement of fishmeal with *L. leucocephala* leaves fermented by *L. plantarum* TISTR1284 in diets for *M. rosenbergii*. Specifically, the study investigated the effects of dietary inclusion of fermented *L. leucocephala* on antioxidant properties of the diets, total hemocyte count, hematopoietic tissue proliferation, phenoloxidase activity, immune-related gene expression, intestinal morphology, and resistance to *A. hydrophila* infection. The findings provide insight into the potential application of fermented *L. leucocephala* as a sustainable functional feed ingredient capable of reducing dependence on fishmeal while enhancing immune competence and disease resistance in freshwater prawn aquaculture.

## MATERIALS AND METHODS

### Ethical approval

All experimental procedures involving giant freshwater prawns (*M. rosenbergii*) were reviewed and approved by the Laboratory Animals Research Center, University of Phayao, and the Animal Ethics Committee of the University of Phayao, Thailand (Approval No. 63 01 04 010; approved on 4 August 2020). The study was conducted in accordance with institutional guidelines for the care and use of aquatic animals in research and relevant standards for animal welfare, biosafety, and humane experimental practice.

Healthy adult prawns were acclimatized before the experiment and maintained under controlled water quality conditions to minimize stress. Animal handling, dietary intervention, bacterial challenge with *A. hydrophila*, hemolymph collection, tissue sampling, and survival monitoring were performed only by trained personnel using standardized procedures. The number of prawns used was limited to the minimum required to achieve the study objectives, and the sample size was determined before experimentation using G*Power software. During the challenge experiment, animals were monitored regularly for abnormal behavior, morbidity, and mortality. All efforts were made to reduce pain, distress, and unnecessary handling throughout the study. Moribund prawns and dead animals were promptly removed from the tanks, and biological waste and bacterial cultures were handled and disposed of in accordance with institutional biosafety procedures.

### Study period and location

The study was conducted from March to May 2021 at the laboratory and aquaculture research facilities of the University of Phayao, Phayao, Thailand. Healthy adult *M. rosenbergii* were obtained from a local commercial farm in Chiang Rai, Thailand, and transported to the laboratory for acclimatization before the feeding trial and bacterial challenge experiment.

### Study design

This study evaluated the effects of partial replacement of fishmeal with fermented *L. leucocephala* on immune responses and resistance to *A. hydrophila* infection in adult *M. rosenbergii*. Three dietary treatments were prepared: a control diet, a diet in which 10% of fishmeal was replaced with fermented *L. leucocephala*, and a diet in which 20% of fishmeal was replaced with fermented *L. leucocephala*. After acclimatization, prawns were randomly allocated to dietary groups and fed for 2 weeks before challenge with *A. hydrophila*. Hemolymph, hematopoietic tissue, and intestinal samples were collected at predetermined time points for immunological, histological, and molecular analyses. A separate survival experiment was conducted using three independent replicate tanks per treatment group.

### *Macrobrachium rosenbergii* culture

Healthy *M. rosenbergii* weighing 25–30 g were obtained from a local farm in Chiang Rai, Thailand, and maintained in the laboratory under acclimatized conditions for 7 days before the start of the experiment. Adult prawns were selected because the present study primarily focused on immune status and immune-related responses following dietary supplementation and bacterial challenge. In addition, their larger body size facilitated the collection of hemolymph for immunological and molecular analyses. During acclimatization, prawns were fed a commercial diet three times daily at 07:00, 13:00, and 19:00 h.

### Bacterial culture

The *A. hydrophila* culture and the protocol for determining the median lethal dose in *M. rosenbergii* were described previously [20]. The stock culture of *L. plantarum* TISTR1284 was stored in the laboratory at −80°C in de Man, Rogosa, and Sharpe medium (HiMedia Laboratories Pvt. Ltd., Mumbai, India) containing 20% glycerol. The thawed stock culture was cultivated in fresh medium and incubated at 37°C for 48 h. The culture was harvested, and the optical density at 600 nm was adjusted to 0.1 using 0.85% sterile NaCl solution to obtain an inoculum concentration of 1.5 × 10⁸ CFU/mL.

### Preparation of fermented *L. leucocephala*

*L. leucocephala* leaf tips were collected, washed, and air-dried. Fermentation was performed using *L. plantarum* TISTR1284 at an inoculum level of 1 × 10⁶ CFU/g plant material following a modified method described previously [21]. The plant material was packed in airtight containers and incubated under anaerobic conditions at room temperature (25°C–30°C) for 30 days. After fermentation, samples were oven-dried and ground into a fine powder for diet formulation. Amino acid composition of fermented and non-fermented samples was analyzed by Central Laboratory (Thailand) Co., Ltd., Bangkok, Thailand, using high-performance liquid chromatography, and the results were expressed as mg/100 g dry weight (Table 1).

**Table 1:** Amino acid composition of fermented and non-fermented Leucaena leucocephala.

**Amino acid**	**Non-fermented ** ***L. leucocephala*** ** (mg/100 g)**	**Fermented ** ***L. leucocephala*** ** (mg/100 g)**
Toxic non-protein amino acid		
Mimosine	92.46	53.46
Essential amino acids		
Phenylalanine	2309	2973
Histidine	1658	1680
Leucine	2291	2829
Lysine	5695	7092
Methionine	<20	<20
Valine	1063	1236
Threonine	248	262
Non-essential amino acids		
Aspartic acid	1026	1216
Glutamic acid	1413	1340
Alanine	678	814
Cystine	246	320
Glycine	479	510
Hydroxyproline	<20	<20
Hydroxylysine	<20	<20
Proline	546	641
Serine	383	903
Isoleucine	1183	1374
Tyrosine	2116	2152
Tryptophan	248	245

Amino acid composition was determined by Central Laboratory (Thailand) Co., Ltd., Bangkok, Thailand, using the in-house method TE-CH-372 based on the Official Journal of the European Communities, Commission Directive 98/64/EC, Annex Part A, L257 (1998), pp. 14–23. Values are expressed as mg/100 g dry weight.

Antioxidant and phytochemical activities were analyzed as shown in Table 2. Fermentation increased most essential and non-essential amino acids while reducing mimosine content, indicating improved nutritional quality of *L. leucocephala* leaves (Table 1).

### Experimental diets

Three diets were prepared for this study. The control diet was formulated using fishmeal as the primary protein source, whereas the two experimental diets incorporated fermented *L. leucocephala* powder to replace 10% or 20% of fishmeal, designated as the 10% *L. leucocephala* and 20% *L. leucocephala* diets, respectively. The dry ingredients were homogenized and subsequently combined with 30% water to form a uniform mash. This mixture was processed into pellets using an extruder (EXT15HP3V03; Siam Farm Services Co., Ltd., Bangkok, Thailand) fitted with a 2-mm die. The pellets were dried at 4°C under forced-air circulation until the moisture content was reduced to approximately 10%. Once dried, all diets were sealed and stored at −20°C until further use.

The ingredient composition of the control and experimental diets is summarized in Table 3. The experimental diets were formulated to evaluate the practical replacement of fishmeal with fermented *L. leucocephala* powder and were not designed to be strictly isonitrogenous or isolipidic.

**Table 2:** Antioxidant and phytochemical activities of non-fermented and fermented Leucaena leucocephala.

**Sample**	**DPPH IC₅₀ (** **μg** **/mL)**	**ABTS⁺ IC₅₀ (** **μg** **/mL)**	**FRAP (mg ** **FeSO** **₄/g extract)**	**TPC (mg GAE/g extract)**	**TFC (mg CE/g extract)**
Non-fermented *L. leucocephala*	458.49 ± 3.26ᵃ	152.60 ± 2.88ᵃ	419.61 ± 24.39ᵃ	171.50 ± 5.44ᵃ	17.429 ± 1.12ᵃ
Fermented *L. leucocephala*	984.12 ± 12.23ᵇ	142.19 ± 1.74ᵃ	259.84 ± 16.09ᵇ	163.56 ± 7.33ᵃ	12.336 ± 0.77ᵇ

Values are presented as mean ± SD (n = 3). Different superscript letters within the same column indicate significant differences between treatments (p < 0.05). ABTS = 2,2′-azinobis-(3-ethylbenzothiazoline-6-sulfonic acid); CE = catechin equivalent; DPPH = 2,2-diphenyl-1-picrylhydrazyl; FRAP = ferric reducing antioxidant power; GAE = gallic acid equivalent; IC₅₀ = half-maximal inhibitory concentration; SD = standard deviation; TFC = total flavonoid content; TPC = total phenolic content.

**Table 3:** Ingredient and proximate composition of experimental diets.

**Item**	**Basal diet**	**10% fermented ** ***L. leucocephala***	**20% fermented ** ***L. leucocephala***
Ingredients (%)			
Fish meal	35	25	15
Fermented *L. leucocephala* leaves with *L. plantarum* TISTR1284	0	10	20
Shrimp shell meal	14	14	14
Soybean meal	25	25	25
Wheat meal	5	5	5
Squid oil	4	4	4
Corn grain	5	5	5
Rice bran	10	10	10
Vitamin and mineral premix	2	2	2
Proximate composition (%)			
Crude protein*	44.63 ± 0.18ᵃ	41.14 ± 0.44ᵇ	36.75 ± 0.28ᶜ
Crude lipid*	4.79 ± 0.32ᵇ	4.85 ± 0.28ᵇ	5.95 ± 0.07ᵃ
Ash*	16.26 ± 0.57ᵃ	13.75 ± 0.39ᵇ	12.37 ± 0.18ᶜ
Carbohydrate*	12.81 ± 0.27ᵃ	17.43 ± 0.98ᵇ	23.14 ± 0.75ᶜ
Fiber*	12.46 ± 0.46ᵇ	14.14 ± 0.14ᵃ	12.37 ± 0.18ᵇ
Moisture*	9.05 ± 0.15ᵃ	8.69 ± 0.32ᵃ	8.01 ± 0.11ᵇ

Proximate composition was analyzed by the School of Agriculture and Natural Resources, University of Phayao. Values are presented as mean ± SD. Different superscript letters within the same row indicate significant differences among experimental diets as determined by one-way analysis of variance followed by Tukey’s multiple comparison test (p < 0.05). *L. leucocephala* = *Leucaena leucocephala*; *L. plantarum* = *Lactiplantibacillus plantarum*; SD = standard deviation.

Following diet preparation, pellet samples were analyzed for amino acid and fatty acid profiles by Central Laboratory (Thailand) Co., Ltd., and the results are presented in Tables 4 and 5.

### Proximate composition analysis

Proximate composition analysis of the experimental diets, including crude protein, crude lipid, ash, moisture, crude fiber, and carbohydrate contents, was conducted according to standard procedures described by the Association of Official Analytical Chemists [22]. Crude protein was determined using the Kjeldahl method, crude lipid by Soxhlet extraction, moisture by oven drying, and ash by combustion in a muffle furnace. Carbohydrate content was calculated by difference using the following formula:

% Carbohydrate = 100 − (% Moisture + % Fat + % Ash + % Crude fiber + % Protein)

The results of proximate composition analysis are shown in Table 3.

### Total phenolic content (TPC)

TPC of fermented and non-fermented *L. leucocephala* leaf powder and experimental diets was analyzed using the Folin–Ciocalteu colorimetric method with modifications [23]. Briefly, 100 µL of each extract was mixed with 125 µL of Folin–Ciocalteu reagent, followed by the addition of 300 µL of 20% sodium carbonate solution. The reaction mixture was adjusted to a final volume of 1 mL with double-distilled water and incubated at 25–30°C in the dark for 2 h. Absorbance was measured at 760 nm using a UV–Vis spectrophotometer. Gallic acid was used as the calibration standard, and the results were expressed as mg GAE/g extract.

**Table 4:** Amino acid composition (mg/100 g diet) of experimental diets supplemented with fermented Leucaena leucocephala at different inclusion levels.

**Amino acid**	**0% fermented ** ***L. leucocephala*** ** (mg/100 g)**	**10% fermented ** ***L. leucocephala*** ** (mg/100 g)**	**20% fermented ** ***L. leucocephala*** ** (mg/100 g)**
Essential amino acids			
Phenylalanine	1773.36	1764.72	1590.50
Histidine	976.49	963.31	831.25
Leucine	3065.22	3059.89	2694.95
Lysine	2711.91	2602.23	2132.84
Methionine	758.82	697.70	538.76
Valine	1911.37	1915.39	1730.93
Threonine	1756.02	1721.07	1489.27
Non-essential amino acids			
Aspartic acid	4654.87	4591.17	4052.13
Glutamic acid	6896.02	6785.67	5705.99
Alanine	2413.33	2358.42	2013.82
Cystine	421.18	401.04	344.95
Glycine	2471.71	2362.73	2003.59
Hydroxyproline	<500.00	<500.00	<500.00
Hydroxylysine	ND	ND	ND
Proline	1885.07	1891.33	1644.76
Serine	1916.24	1890.40	1650.81
Isoleucine	1655.99	1653.26	1467.55
Tyrosine	1496.31	1468.86	1263.05
Tryptophan	387.04	379.41	322.55

Amino acid composition was determined by Central Laboratory (Thailand) Co., Ltd. using in-house method TE-CH-372 based on Official Journal of the European Communities, Commission Directive 98/64/EC, Annex Part A, L257 (1998), pp. 14–23. ND = not detected; *L. leucocephala* = *Leucaena leucocephala*.

### Total flavonoid content (TFC)

TFC of fermented and non-fermented *L. leucocephala* leaf powder and experimental diets was determined using the aluminum chloride colorimetric method [23]. Briefly, 250 µL of extract was mixed with 75 µL of 5% sodium nitrite solution and incubated for 6 min. Then, 150 µL of 10% aluminum chloride solution was added, and the mixture was incubated for 5 min. Subsequently, 500 µL of 1 M sodium hydroxide was added, and the volume was adjusted to 2.5 mL with distilled water. Absorbance was measured at 510 nm using a UV–Vis spectrophotometer. Catechin was used as the reference standard, and the results were expressed as mg CE/g extract.

### Evaluation of antioxidant activities

**DPPH radical scavenging activity:** Fermented and non-fermented *L. leucocephala* leaf powder and experimental diets were evaluated using the DPPH radical scavenging assay [23]. DPPH solution was prepared in ethanol and protected from light before use. The extracts were diluted in ethanol at different concentrations (0–20 mg/mL). Twenty microliters of sample solution was combined with 180 µL of DPPH solution in a 96-well microplate, then incubated in the dark at 37°C for 30 min. Absorbance was read at 540 nm using a microplate reader. Ascorbic acid and Trolox were used as positive controls, whereas DPPH solution without sample was used as the negative control. Radical scavenging activity was expressed as percentage inhibition, and IC_50_ values were obtained by nonlinear regression analysis using GraphPad Prism version 10.5.0.774 (GraphPad Software, Boston, MA, USA).

**ABTS radical scavenging activity:** ABTS radical scavenging activity of fermented and non-fermented *L. leucocephala* leaf powder and experimental diets was determined using a previously reported method with modifications [23]. Briefly, 20 µL of extract at various concentrations (0–20 mg/mL) was mixed with 2.0 mL of diluted ABTS working solution. The ABTS radical cation was generated by mixing 7.5 mM ABTS stock solution with 2.5 mM potassium persulfate in the dark at 25°C–30°C for 16 h. The reaction mixtures were incubated for 5 min at 25°C, and absorbance was measured at 734 nm using a UV–Vis spectrophotometer (Optizen POP, Mecasys Co., Ltd., Daejeon, Korea). Percentage inhibition and IC_50_ values were calculated using dose-response curves generated with GraphPad Prism software.

**Table 5:** Fatty acid composition of the experimental diets.

**Fatty acid**	**0% fermented ** ***L. leucocephala*** ** (g/100 g)**	**10% fermented ** ***L. leucocephala*** ** (g/100 g)**	**20% fermented ** ***L. leucocephala*** ** (g/100 g)**
Lauric acid	0.02	0.02	0.02
Myristic acid	0.13	0.13	0.12
Pentadecanoic acid	0.04	0.04	0.04
Palmitic acid	1.50	1.80	2.02
Heptadecanoic acid	0.05	0.05	0.04
Stearic acid	0.44	0.53	0.57
**Saturated fatty acids**	**2.21**	**2.60**	**2.85**
Palmitoleic acid	0.16	0.14	0.11
Cis-9-oleic acid	1.79	1.65	1.49
Cis-11-eicosenoic acid	0.09	0.15	0.17
Erucic acid	0.04	0.03	0.03
Nervonic acid	0.02	0.02	0.03
**Monounsaturated fatty acids**	**2.11**	**2.02**	**1.85**
Cis-9,12-linoleic acid	0.82	0.84	0.68
Gamma-linolenic acid	0.03	0.04	0.05
Alpha-linolenic acid	0.04	0.04	0.04
Cis-11,14-eicosadienoic acid	0.01	0.01	ND
Cis-8,11,14-eicosatrienoic acid	0.03	0.03	0.03
Cis-11,14,17-eicosatrienoic acid	0.05	0.04	0.03
Cis-13,16-docosadienoic acid	0.01	ND	ND
Cis-5,8,11,14,17-eicosapentaenoic acid	0.05	0.05	0.02
4,7,10,13,16,19-docosahexaenoic acid	0.08	0.09	0.03
**Polyunsaturated fatty acids**	**1.12**	**1.15**	**0.88**
**Unsaturated fatty acids**	**3.23**	**3.17**	**2.73**
Trans fat	0.01	0.02	0.02

Fatty acid composition was determined by Central Laboratory (Thailand) Co., Ltd. using in-house method TE-CH-208 based on AOAC (2019) Method 996.06. ND = not detected; *L. leucocephala* = *Leucaena leucocephala*.

### FRAP assay

The reducing ability of fermented and non-fermented *L. leucocephala* leaf powder and experimental diets was determined using the FRAP assay [24]. The assay is based on the reduction of ferric ions to ferrous ions, forming a blue Fe²⁺/TPTZ complex. The extract was mixed with freshly prepared FRAP reagent and incubated at 25°C–30°C for 30 min. Absorbance was recorded at 593 nm using a UV–Vis spectrophotometer. Antioxidant activity was expressed as mg FeSO₄/g extract.

### Experimental challenge and sampling

To evaluate whether replacing fishmeal protein with fermented *L. leucocephala* leaves affects the immune system of prawns, adult prawns were selected because they possess a fully developed immune system, allowing consistent and reliable evaluation of diet-induced immune responses. For the experimental challenge and immune assessment, prawns were acclimatized for 1 week, during which they were fed the control diet three times daily at 07:00, 13:00, and 19:00 h. They were then randomly divided into three groups (n = 70 per group) and maintained in plastic tanks (44 × 62 × 34 cm) containing 45 L of aerated water, with 15 prawns per tank.

Sample size determination was performed using G*Power software version 3.1.9.7 prior to experimentation to ensure adequate statistical power to detect differences among treatment groups. Group 1 received the basic diet and served as the control group. Group 2 received the basic diet with 10% fishmeal replaced by fermented *L. leucocephala*. Group 3 received the basic diet with 20% fishmeal replaced by fermented *L. leucocephala*. Prawns were fed three times daily at 07:00, 13:00, and 19:00 h for 2 weeks.

After the 2-week feeding period, prawns were injected with *A. hydrophila* at a dose corresponding to the previously reported median lethal dose for *M. rosenbergii* (8.91 × 10⁵ CFU/mL) [20] and maintained under the same feeding regimen and environmental conditions for 1 week. At each sampling time point (6, 12, 24, 48, 72, 96, and 120 h post-infection), five prawns were randomly selected from each treatment group for collection of hemolymph, hematopoietic tissue, and intestinal samples for immunological analyses. This experiment was conducted using a single replicate per treatment group. For the survival experiment, prawns were divided into three groups as described above, with n = 15 per group. This experiment was conducted using three independent replicate tanks per treatment group to observe and record mortality.

### Water quality parameters

Water quality parameters were monitored throughout the experimental period. Water temperature was maintained at 27°C–29°C, DO above 5 mg/L, and pH at 7.5–8.0. Total ammonia and nitrite concentrations were maintained below 0.1 mg/L and 0.5 mg/L, respectively, which are within acceptable ranges for *M. rosenbergii* culture [25]. To maintain water quality, approximately 30%–50% of the water was replaced every 3 days, and the filtration system was operated continuously throughout the experiment.

### Total hemocyte count

Hemolymph (500 µL) was withdrawn from the heart of *M. rosenbergii* into a 1-mL syringe containing cold Alsever’s solution and transferred to a 1.5-mL microcentrifuge tube. A drop of mixed hemolymph was placed on a hemocytometer, and total hemocyte count was determined under a light microscope (Olympus CX22; Olympus Corporation, Tokyo, Japan). Hemocyte counting was performed by an investigator blinded to the treatment groups.

### PO activity

PO activity in hemolymph was measured according to a previously described protocol with modifications [26]. Briefly, 100 µL of hemolymph was withdrawn from the heart into a 1-mL syringe containing 900 µL of cold Tris-buffered saline-I (50 mM Tris, 210 mM NaCl, 5 mM KCl, and 2.5 mM MgCl₂, pH 7.5). The hemolymph was centrifuged at 5000 × *g* for 15 min at 4°C to separate hemocytes, and the resulting hemolymph supernatant was collected for PO activity analysis. Ten microliters of hemolymph were incubated with 190 µL of 5 mM L-DOPA (D9628; Sigma-Aldrich, St. Louis, MO, USA) dissolved in 50 mM Tris-HCl, pH 7.5, for 20 min at 25°C to develop dopachrome. The dopachrome reaction was measured at 490 nm using a VersaMax™ Microplate Reader (Molecular Devices, San Jose, CA, USA).

### Histology

Hematopoietic tissue was collected, fixed in Davison’s fixative for 24 h, and then washed with 70% ethanol. The tissue was processed using an automatic sample preparation system (Tissue-Tek® VIP™ 5 Jr.; Sakura Finetek Japan Co., Ltd., Tokyo, Japan) and embedded in paraffin. Paraffin blocks were sectioned at 5 µm, and the sections were placed on glass slides. Tissue sections were stained with Mayer’s hematoxylin (Cat. No. 05-06002/L; Bio-Optica Milano SpA, Milan, Italy) and eosin Y plus alcoholic solution (Cat. No. 05-11007/L; Bio-Optica Milano SpA). Hematopoietic tissue sections were examined under a light microscope (Nikon Upright Microscope Eclipse Ni-U; Nikon Corporation, Tokyo, Japan) at 40× magnification. Prophase, metaphase, anaphase, and telophase in hematopoietic cell nuclei were counted and averaged. Mitotic cell quantification was performed by an investigator blinded to the treatment groups.

### Quantitative real-time polymerase chain reaction (PCR)

Total RNA was isolated from various tissues of *M. rosenbergii* using Tri Reagent® (Cat. No. TR118; Molecular Research Center, Inc., Cincinnati, OH, USA) according to the manufacturer’s protocol. RNA quality and quantity were assessed by measuring absorbance at 260 and 280 nm using NanoDrop One (Thermo Fisher Scientific, Waltham, MA, USA). One microgram of RNA was subjected to cDNA synthesis using ReverTra Ace™ quantitative (q)PCR RT Master Mix with gDNA Remover (TOYOBO Co., Ltd., Osaka, Japan) according to the manufacturer’s protocol. The cDNA was stored at −20°C until further analysis.

The cDNA samples were analyzed for relative expression levels of the genes anti-lipopolysaccharide factor (*ALF*), Crustacean hematopoietic factor (*CHF*), *proPO*, *Relish*, *TRAF6*, *Dorsal*, *IMD*, *HSP70*, and *Cu/Zn-SOD* in hemocytes and hematopoietic tissue using QIAquant 96 5plex (Qiagen, Hilden, Germany). Amplification was performed in a 96-well plate with a 20-µL fluorescent qPCR reaction mixture. The reaction mixture contained 1 µL of cDNA, 10 µL of SensiFAST™ SYBR® No-ROX kit (BIO-98050; Meridian Bioscience, Cincinnati, OH, USA), 0.4 µL each of forward and reverse primers (10 µM/µL), and 8.2 µL of sterile dH₂O. All qRT-PCR primers are listed in Table 6[27–32].

**The thermal qRT-PCR cycle profile followed the manufacturer’s protocol:** one cycle of enzyme activation at 95°C for 2 min, followed by 40 cycles of denaturation at 95°C for 5 s and annealing/extension at 60°C for 20 s. Sterile dH₂O served as the negative control. *EF-1α* was selected as the reference gene for normalization. After completion of the qRT-PCR program, data were analyzed using QIAquant96 software. The baseline was set automatically by the software to maintain consistency. The comparative CT (2−ΔΔCT) method was used to analyze gene expression levels [33].

**Table 6:** Nucleotide sequences of primers used in this study.

**Gene**	**Primer sequence (5′–3′)**	**Accession No.**	**Amplicon size (bp)**	**Reference**
ALF	F: GTCTTGGGTTGTTTTGGTAA	*	103	[27]
	R: CATCGTTACTTCCCACTTGT			
CHF	F: GAGGGTCTGTCTTGCTACTG	MH595490.1	220	[27]
	R: GGTACTTCTCCTCGTCTCCT			
C-lectin	F: ACTCTGTTGGACAACTCCAC	*	248	[28]
	R: ACCCGGAGAGAAAGTAAGAC			
Relish	F: GATGAGCCTTCAGTGCCAGA	KR827675.1	240	[29]
	R: CCAGGTGACGCCATGTATCA			
IMD	F: CGACCACATTCTCCTCCTCCC	MT123546.1	220	[29]
	R: TTCAGTGCATCCACGTCCCTC			
Dorsal	F: TCAGTAGCGACACCATGCAG	KX219631.1	360	[29]
	R: CGAGCCTTCGAGGAACACTT			
TRAF6	F: TCTGGATTGTGGTCCCACTG	MH507502.1	117	[30]
	R: ATGGGTCGCTGAAATGCTTG			
proPO	F: ACTCTTCCATCACTGCACCG	DQ182596.1	360	[31]
	R: CCTGCCTCGGATGACTTGTT			
HSP70	F: GTCCTGATGAAGATGAAGGA	MH846234.1	360	[31]
	R: CCTTGCCACTTGTTACTTTC			
Cu/Zn-SOD	F: TCGCCTAACGAGGAGGTTC	DQ121374.1	81	[32]
	R: CGGCTTCATCAGGATTTTGAG			
EF-1α	F: ATGTCATGGTGGAAGAAGAG	KF228019.1	360	[27]
	R: AAAGTTGACCACCATACCAG			

The ALF and C-lectin primers were designed based on sequencing analysis from a previous study [27] and have been previously used and reported by Jariyapong *et al.* [27] and Pudgerd *et al.* [28]. ALF = anti-lipopolysaccharide factor; CHF = crustacean hematopoietic factor; Cu/Zn-SOD = copper/zinc superoxide dismutase; EF-1α = elongation factor-1 alpha; HSP70 = heat shock protein 70; IMD = immune deficiency; proPO = prophenoloxidase; TRAF6 = tumor necrosis factor receptor-associated factor 6.

### Statistical analysis

Data are presented as mean ± standard deviation. Statistical analyses were performed using GraphPad Prism software version 10.5.0.774 (GraphPad Software, Boston, MA, USA). Before statistical analysis, data normality was assessed using the Shapiro–Wilk test, and homogeneity of variance was evaluated using the Brown–Forsythe test. Data that satisfied parametric assumptions were analyzed using one-way analysis of variance, followed by Tukey’s multiple comparisons test. When assumptions of normality or homogeneity of variance were not met, non-parametric analysis was performed using the Kruskal–Wallis test followed by Dunn’s multiple comparison test. Survival data were analyzed using the Kaplan–Meier method. Differences were considered statistically significant at p < 0.05.

## RESULTS

### Amino acid profile of fermented *L. leucocephala*

Fermentation of *L. leucocephala* significantly altered the amino acid profile. Most essential amino acids increased after fermentation, including lysine (+24.6%), leucine (+23.5%), phenylalanine (+28.7%), and valine (+16.3%). In contrast, the toxic non-protein amino acid mimosine was markedly reduced by 42.2%, indicating detoxification during fermentation (Table 1). Overall, the total essential amino acid content was higher in the fermented material than in the non-fermented material.

The inclusion of fermented *L. leucocephala* meal gradually altered the amino acid composition of the diets in a dose-dependent manner (Table 4). Most essential amino acids, including lysine, methionine, leucine, and threonine, showed a decreasing trend as the inclusion level increased from 0% to 20%. Lysine content decreased from 2711.91 mg/100 g in the control diet to 2132.84 mg/100 g in the 20% replacement group. A similar pattern was observed for methionine and threonine. Non-essential amino acids also showed a general decreasing trend with increasing inclusion levels, particularly glutamic acid and aspartic acid. Overall, dietary amino acid profiles were moderately affected by higher inclusion levels of fermented leaf meal.

**Antioxidant and phytochemical properties of experimental diets supplemented with fermented** *L. leucocephala*

The antioxidant activity and phytochemical contents of diets supplemented with fermented *L. leucocephala* are shown in Table 7. Antioxidant capacity and phytochemical content increased dose-dependently with increasing levels of fermented *L. leucocephala* supplementation. The 20% fermented *L. leucocephala* diet showed the highest antioxidant activity in both DPPH and ABTS radical scavenging assays, as evidenced by the lowest IC_50_ values (1612.57 ± 19.87 and 305.38 ± 17.19 µg/mL, respectively). In contrast, the control diet showed the lowest activity. Likewise, FRAP values were significantly higher with increasing supplementation levels, ranging from 6.02 ± 0.50 mg FeSO₄/g extract in the control diet to 27.78 ± 2.23 mg FeSO₄/g extract in the 20% supplementation group. Phytochemical analysis also showed higher TPC and TFC after supplementation with fermented *L. leucocephala*. The highest TPC (11.62 ± 0.50 mg GAE/g extract) and TFC (1.364 ± 0.4 mg CE/g extract) values were observed in the 20% supplementation group compared with the control diet. Overall, the results showed that fermented *L. leucocephala* improved the antioxidant activity and phytochemical profiles of the experimental diets, particularly at the 20% supplementation level.

### Immunological parameters

**Figure 1:**
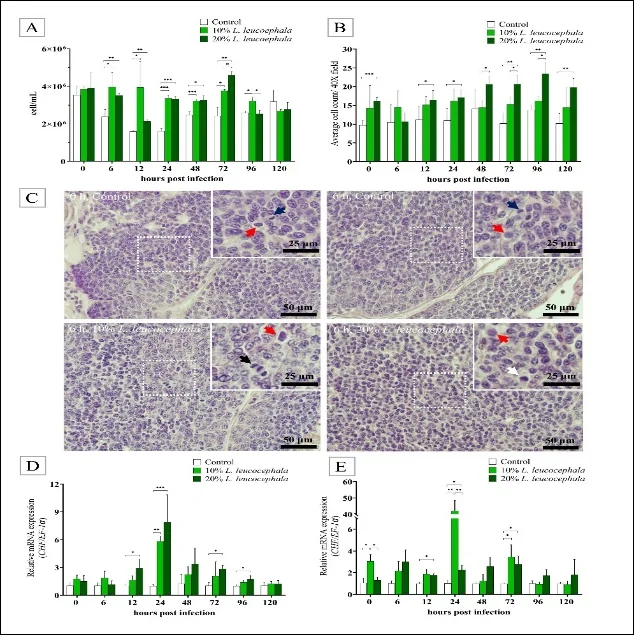
Hemocyte counts and hematopoietic cell proliferation in *Macrobrachium rosenbergii* fed fermented *Leucaena leucocephala* and infected with *Aeromonas hydrophila*. (A) Total hemocyte count at different time points post-infection (n = 5). (B) Average number of mitotic hematopoietic cells at different time points post-infection (n = 5). (C) Representative hematopoietic tissue at 6 h post-infection undergoing cell division; red arrow = prophase, white arrow = metaphase, black arrow = anaphase, and blue arrow = telophase. (D) Relative mRNA expression of CHF in hemocytes. (E) Relative mRNA expression of CHF in hematopoietic tissue. Data are expressed as mean ± SD. For gene expression analyses, n = 3 (cDNA pooled from two prawns per sample). *p < 0.05; **p < 0.01; ***p < 0.001.

**Hemocyte proliferation:** The groups of *M. rosenbergii* fed diets supplemented with 10% and 20% fermented *L. leucocephala* had more circulating hemocytes than the control group and showed a statistically significant increase in circulating hemocytes during 6–72 h after infection with *A. hydrophila* (Figure 1A). At 96 h, only the 10% *L. leucocephala* group (3.22 × 10⁶ ± 1.94 × 10⁵ cells/mL) had significantly higher hemocyte circulation than the control (2.58 × 10⁶ ± 8.7 × 10⁴ cells/mL) and 20% *L. leucocephala* groups (2.54 × 10⁶ ± 1.77 × 10⁵ cells/mL) (p < 0.05, Figure 1A).

Hematopoietic cell division was significantly greater in the 20% *L. leucocephala* group than in the control group at 0 h (p < 0.001), 12–24 h (p < 0.05), and 72–120 h (p < 0.01) post-infection (Figure 1B). Although the 10% *L. leucocephala* group showed a trend toward a higher number of dividing cells, the difference was not statistically significant compared with the 20% *L. leucocephala* group and the control group (Figure 1B). Hematopoietic cells undergoing cell division are shown in Figure 1C and Supplementary Figure S1. Dividing hematopoietic cells were identified by nuclear membrane disappearance and chromosome condensation during prophase (Figure 1C, red arrow), metaphase (Figure 1C, white arrow), anaphase (Figure 1C, black arrow), and telophase (Figure 1C, blue arrow).

The expression of *CHF* in hemocytes at all time points was greater in the 10% and 20% *L. leucocephala* groups than in the control group (Figure 1D). However, a significant increase in *CHF* expression was observed in the 20% *L. leucocephala* group at 12 h (2.92 ± 1.00, p < 0.05), 24 h (7.90 ± 1.00, p < 0.001), 72 h (2.86 ± 0.36, p < 0.05), and 96 h (1.72 ± 0.37, p < 0.05) post-infection compared with the control group. In the 10% *L. leucocephala* group, *CHF* expression (5.83 ± 0.56) was significantly increased at 24 h (p < 0.01) compared with the control group (1.00 ± 0.15). However, no statistically significant difference in hemocyte *CHF* expression was observed between the 10% and 20% *L. leucocephala* groups.

**Figure 2:**
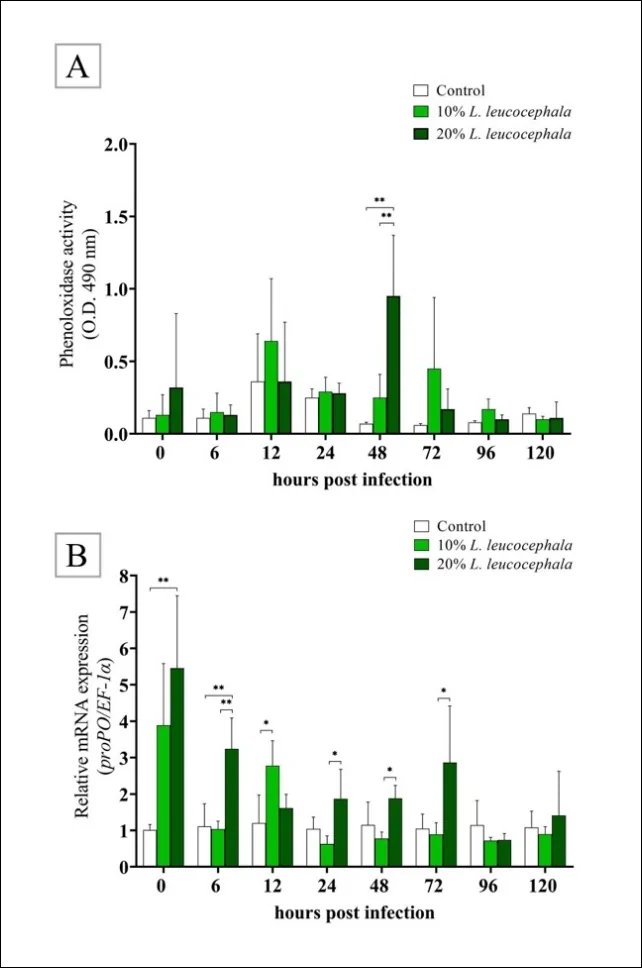
Effects of dietary fermented *Leucaena leucocephala* on phenoloxidase activity and prophenoloxidase expression in *Macrobrachium rosenbergii* infected with *Aeromonas hydrophila*. (A) Phenoloxidase activity in hemolymph. (B) Relative mRNA expression of prophenoloxidase in hemocytes. Data are expressed as mean ± SD. For gene expression analyses, n = 3 (cDNA pooled from two prawns per sample). *p < 0.05; **p < 0.01; ***p < 0.001.

The expression of *CHF* in the hematopoietic tissue of the 10% *L. leucocephala* group was significantly greater at 0 h (3.05 ± 0.60, p < 0.05), 24 h (42.24 ± 6.15, p < 0.01), and 72 h (3.46 ± 1.10, p < 0.05) post-infection compared with the control group (Figure 1E). The expression of *CHF* in the 20% *L. leucocephala* group tended to be higher than that in the control group at 0–120 h post-infection, with significant increases observed only at 12–24 h and 72 h (p < 0.05) (Figure 1E). However, *CHF* expression in the 20% *L. leucocephala* group was consistently lower than that in the 10% *L. leucocephala* group. PO activity: PO activity in the hemolymph of the three treatment groups of *M. rosenbergii* during *A. hydrophila* infection is shown in Figure 2A. Although *M. rosenbergii* receiving 10% and 20% *L. leucocephala* showed a trend toward increased PO activity during 0–96 h post-infection, significantly higher PO activity than that in the control group was observed only at 48 h (p < 0.01).

In addition, mRNA expression of *proPO* in hemocytes significantly increased during 0–72 h post-infection (Figure 2B). The expression of *proPO* in the 20% *L. leucocephala* group was significantly higher than that in the control group at 0–6 h (p < 0.01) and 24–72 h (p < 0.05) post-infection. In the 10% *L. leucocephala* group, *proPO* expression was elevated at 0 h but did not differ significantly from that in the control group; however, it became significantly higher at 12 h post-infection compared with the control group (p < 0.05) (Figure 2B).

### Immune-related gene expression in hemocytes and hematopoietic tissue

**Figure 3:**
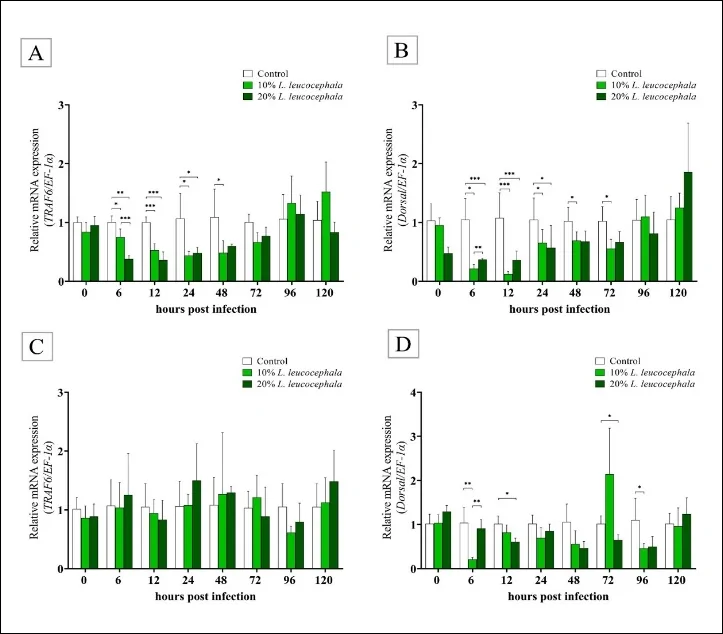
Expression of Toll/Dorsal pathway genes in hemocytes and hematopoietic tissue of *Macrobrachium rosenbergii* infected with *Aeromonas hydrophila*. (A) Relative mRNA expression of TRAF6 in hemocytes. (B) Relative mRNA expression of Dorsal in hemocytes. (C) Relative mRNA expression of TRAF6 in hematopoietic tissue. (D) Relative mRNA expression of Dorsal in hematopoietic tissue. Data are expressed as mean ± SD. For gene expression analyses, n = 3 (cDNA pooled from two prawns per sample). *p < 0.05; **p** <** 0.01; ***p < 0.001.

The mRNA expression levels of *TRAF6* and *Dorsal* in hemocytes were significantly lower in the 10% and 20% *L. leucocephala* groups than in the control group during 6–48 h post-infection (Figure 3A and B). At 72 h, *TRAF6* expression remained downregulated in both treatment groups; however, the differences were no longer statistically significant compared with the control group. *TRAF6* expression subsequently returned to levels comparable to those of the control group at 96–120 h post-infection (Figure 3A). Similarly, *Dorsal* mRNA expression was downregulated in the 10% and 20% *L. leucocephala* groups during 6–72 h and then returned to near control levels at 96–120 h post-infection (Figure 3B).

In hematopoietic tissue, *TRAF6* expression did not differ significantly among the three groups throughout the experimental period (Figure 3C). In contrast, *Dorsal* expression in hematopoietic tissue was significantly decreased at 6 h in the 10% *L. leucocephala* group (0.21 ± 0.04) compared with the 20% *L. leucocephala* group (0.91 ± 0.19) and the control group (1.04 ± 0.34) (p < 0.01, Figure 3D). Although a general trend toward reduced *Dorsal* expression was observed at other time points, significant reductions were detected only in the 20% *L. leucocephala* group at 12 h (0.60 ± 0.08) and 72 h (0.64 ± 0.12), and in the 10% *L. leucocephala* group at 96 h (0.46 ± 0.10), compared with the control group (1.09 ± 0.49) (Figure 3D).

**Figure 4:**
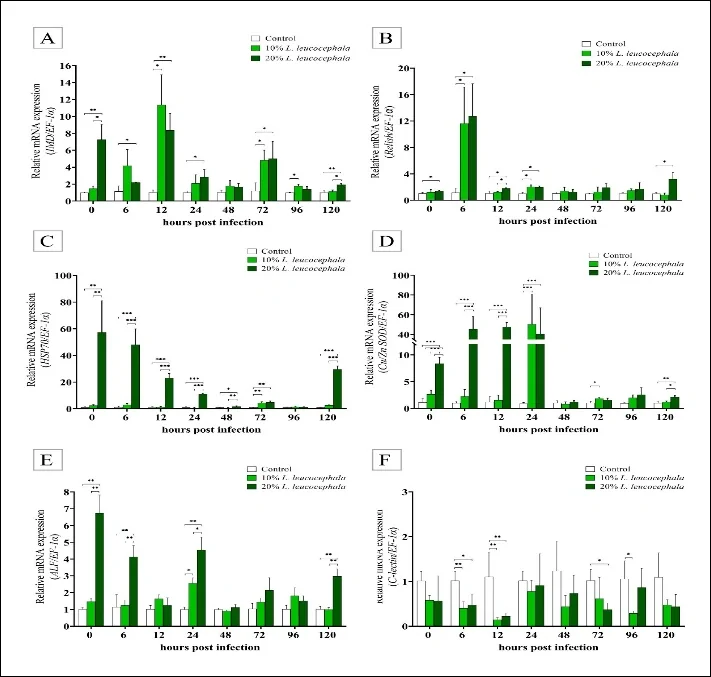
Effects of dietary fermented *Leucaena leucocephala* on IMD/Relish pathway gene expression in hemocytes of *Macrobrachium rosenbergii* infected with *Aeromonas hydrophila*. (A) Relative mRNA expression of IMD. (B) Relative mRNA expression of Relish. (C) Relative mRNA expression of HSP70. (D) Relative mRNA expression of Cu/Zn-SOD. (E) Relative mRNA expression of ALF. (F) Relative mRNA expression of C-lectin. Data are expressed as mean ± SD. For gene expression analyses, n = 4 (cDNA pooled from two prawns per sample). *p < 0.05; **p < 0.01; ***p < 0.001.

Overall, *IMD* expression was elevated in the 10% and 20% *L. leucocephala* groups, although expression patterns varied across time points (Figure 4A). At 0 h, *IMD* expression in hemocytes did not differ significantly between the 10% *L. leucocephala* group and the control group. However, the 20% *L. leucocephala* group (7.25 ± 1.74) showed significantly higher *IMD* expression than both the 10% *L. leucocephala* group (1.46 ± 0.29) (p < 0.05) and the control group (0.99 ± 0.6) (p < 0.01) (Figure 4A). During *A. hydrophila* infection, *IMD* expression remained upregulated in the 10% *L. leucocephala* group, although the increase was not statistically significant compared with the control group. In contrast, the 20% *L. leucocephala* group (2.20 ± 0.01) exhibited significantly higher *IMD* expression at 6 h post-infection than the control group (1.14 ± 0.63) (p < 0.05) (Figure 4A). *IMD* expression further increased in both treatment groups during 12–24 h post-infection, with the highest expression observed at 12 h (Figure 4A). Thereafter, although *IMD* expression remained higher than that in the control group, statistically significant differences were observed only at 72 h in both the 10% and 20% *L. leucocephala* groups (Figure 4A).

*Relish* expression was significantly increased in the 20% *L. leucocephala* group at 0 h (1.40 ± 0.08) compared with the control group (1.01 ± 0.17) (p < 0.05) (Figure 4B). At 6 h after bacterial challenge, *Relish* expression was significantly elevated in both the 10% (11.64 ± 5.49) and 20% *L. leucocephala* groups (12.69 ± 4.93) compared with the control group (1.13 ± 0.67) (p < 0.05) (Figure 4B). At 12 h, significantly higher *Relish* expression was observed only in the 20% *L. leucocephala* group (1.77 ± 0.10) compared with the 10% *L. leucocephala* group (1.23 ± 0.11) and the control group (1.04 ± 0.35) (p < 0.05) (Figure 4B). At 24 h, *Relish* expression was significantly upregulated in the 10% *L. leucocephala* group compared with the control group (p < 0.05) (Figure 4B). Although slight increases in *Relish* expression were observed in both treatment groups during 48–96 h post-infection, the differences were not statistically significant. At 120 h, *Relish* expression was significantly higher in the 20% *L. leucocephala* group (3.19 ± 1.04) than in the control group (1.00 ± 0.17) (p < 0.05) (Figure 4B).

*HSP70* expression was significantly higher in the 20% *L. leucocephala* group than in the 10% *L. leucocephala* and control groups throughout the 0–120 h post-infection period (p < 0.001) (Figure 4C). *HSP70* expression was highest at 0 h and gradually decreased until 48 h post-infection. Thereafter, expression gradually increased during 72–96 h, and by the end of the experimental period, no significant differences in *HSP70* expression were observed among the groups.

*Cu/Zn-SOD* mRNA expression was significantly upregulated in the 20% *L. leucocephala* group compared with the 10% *L. leucocephala* and control groups during 0–12 h and at 120 h post-infection (p < 0.001) (Figure 4D). At 24 h post-infection, *Cu/Zn-SOD* expression was markedly elevated in both the 10% (50.44 ± 29.87) and 20% *L. leucocephala* groups (40.48 ± 26.46) compared with the control group (0.92 ± 0.17), although no significant difference was observed between the two treatment groups. During 72–120 h post-infection, both *L. leucocephala*-supplemented groups showed a trend toward increased *Cu/Zn-SOD* expression relative to the control group.

In addition, the marked upregulation of *Cu/Zn-SOD* expression in the 20% *L. leucocephala* group was associated with significantly lower ABTS and DPPH IC_50_ values than in the control group, indicating enhanced radical-scavenging activity. This group also exhibited higher FRAP values and increased TPC and TFC compared with the control group (Table 7). These findings suggest that elevated antioxidant gene expression corresponded with improved antioxidant capacity and phytochemical content.

**Table 7:** Antioxidant and phytochemical activities of experimental diets.

**Diet**	**DPPH IC₅₀ (** **μg** **/mL)**	**ABTS⁺ IC₅₀ (** **μg** **/mL)**	**FRAP (mg ** **FeSO** **₄/g extract)**	**TPC (mg GAE/g extract)**	**TFC (mg CE/g extract)**
0% fermented *L. leucocephala*	6,436.66 ± 88.19ᵃ	1,604.48 ± 51.51ᵃ	6.02 ± 0.50ᵃ	2.40 ± 0.15ᵃ	−0.134 ± 0.1ᵃ
10% fermented *L. leucocephala*	2,189.23 ± 52.06ᵇ	496.55 ± 12.64ᵇ	15.13 ± 2.06ᵇ	7.88 ± 0.42ᵇ	0.882 ± 1.2ᵃ
20% fermented *L. leucocephala*	1,612.57 ± 19.87ᶜ	305.38 ± 17.19ᶜ	27.78 ± 2.23ᶜ	11.62 ± 0.50ᶜ	1.364 ± 0.4ᵃ

Values are presented as mean ± SD (n = 3). Different superscript letters within the same column indicate significant differences among treatments (p < 0.05). ABTS = 2,2′-azinobis-(3-ethylbenzothiazoline-6-sulfonic acid); CE = catechin equivalent; DPPH = 2,2-diphenyl-1-picrylhydrazyl; FRAP = ferric reducing antioxidant power; GAE = gallic acid equivalent; IC₅₀ = half-maximal inhibitory concentration; SD = standard deviation; TFC = total flavonoid content; TPC = total phenolic content; *L. leucocephala* = *Leucaena leucocephala*.

*ALF* mRNA expression was generally upregulated in the 10% and 20% *L. leucocephala* groups compared with the control group throughout the 0–120 h post-infection period (Figure 4E). The 20% *L. leucocephala* group exhibited significantly higher *ALF* expression than both the 10% *L. leucocephala* and control groups at 0 h (p < 0.01), 6 h (p < 0.01), 24 h (p < 0.05 and p < 0.01, respectively), and 120 h post-infection (p < 0.01) (Figure 4E). In contrast, *ALF* expression in the 10% *L. leucocephala* group (2.55 ± 0.32) was significantly higher than that in the control group (1.00 ± 0.12) only at 24 h post-infection (p < 0.05) (Figure 4E). In contrast to *ALF*, *C-lectin* expression was generally lower in the 10% and 20% *L. leucocephala* groups than in the control group at all time points. However, significant reductions were observed only during 6–12 h and 72–96 h post-infection (Figure 4F).

### Intestinal morphology and intestinal immune-related gene expression

**Figure 5:**
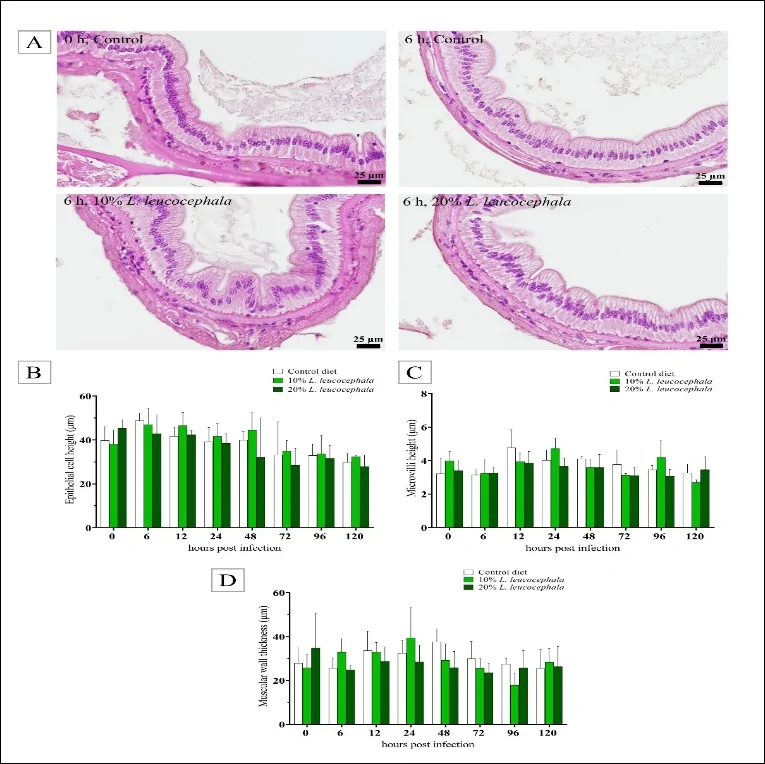
Effects of dietary fermented *Leucaena leucocephala* on intestinal morphology of *Macrobrachium rosenbergii* infected with *Aeromonas hydrophila*. (A) Representative hematoxylin and eosin-stained intestinal sections. (B) Epithelial cell height. (C) Microvillus height. (D) Muscular wall thickness. Data are expressed as mean ± SD (n = 5).

The histology of *M. rosenbergii* intestines showed normal structures in all groups (Figure 5A and Supplementary Figure S2). Alterations in epithelial cell height (Figure 5B), microvilli height (Figure 5C), and muscular wall thickness (Figure 5D) were not observed.

**Figure 6:**
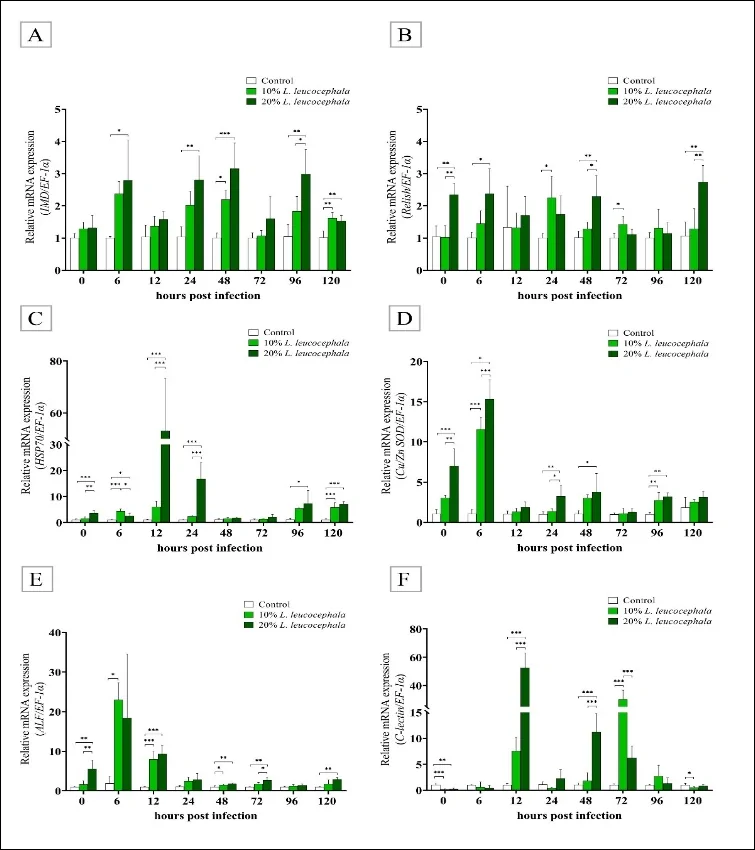
Effects of dietary fermented *Leucaena leucocephala* on IMD/Relish pathway and immune-related gene expression in the intestine of *Macrobrachium rosenbergii* infected with *Aeromonas hydrophila*. (A) Relative mRNA expression of IMD. (B) Relative mRNA expression of Relish. (C) Relative mRNA expression of HSP70. (D) Relative mRNA expression of Cu/Zn-SOD. (E) Relative mRNA expression of ALF. (F) Relative mRNA expression of C-lectin. Data are expressed as mean ± SD. For gene expression analyses, n = 4 (cDNA pooled from two prawns per sample). *p < 0.05; **p < 0.01; ***p < 0.001.

Overall, *IMD* expression was higher in the 20% *L. leucocephala* group than in the control and 10% *L. leucocephala* groups throughout the experimental period (Figure 6A). Compared with the control group, *IMD* expression in the 20% *L. leucocephala* group was significantly increased at 6 h (p < 0.05), 24 h (p < 0.001), and 48 and 96 h post-infection (p < 0.01), and at 120 h post-infection (p < 0.01). However, *IMD* expression in the 20% *L. leucocephala* group did not differ significantly from that in the 10% *L. leucocephala* group. In the 10% *L. leucocephala* group, *IMD* expression was significantly higher than that in the control group at 48 h (p < 0.05) and 120 h post-infection (p < 0.01) (Figure 6A).

*Relish* expression (Figure 6B) was generally higher in the 20% *L. leucocephala* group than in the control and 10% *L. leucocephala* groups throughout the experimental period. Specifically, *Relish* expression in the 20% *L. leucocephala* group was significantly elevated (p < 0.01) at 0 h (2.34 ± 0.34) compared with the control (1.04 ± 0.32) and 10% *L. leucocephala* groups (1.02 ± 0.35). Significant increases were also observed at 6 h (2.37 ± 0.77) compared with the control group (1.01 ± 0.17) and 10% *L. leucocephala* group (1.28 ± 0.21), at 48 h (2.29 ± 0.64) compared with both the control (1.01 ± 0.19) and 10% *L. leucocephala* groups (1.28 ± 0.21) (p < 0.01 and p < 0.05, respectively), and at 120 h (2.74 ± 0.50) compared with the control group (1.06 ± 0.43) and 10% *L. leucocephala* group (1.28 ± 0.62) (p < 0.01) post-infection. Although *Relish* expression in the 10% *L. leucocephala* group tended to be higher than that in the control group, significant differences were observed only at 24 h (2.25 ± 0.66) and 72 h (1.42 ± 0.25) post-infection (p < 0.05) (Figure 6B).

*HSP70* expression (Figure 6C) was generally higher in the 20% *L. leucocephala* group than in the control and 10% *L. leucocephala* groups throughout the experimental period. At 0 h, *HSP70* expression in the 20% *L. leucocephala* group (3.62 ± 0.84) was significantly higher than that in the control (1.03 ± 0.31) and 10% *L. leucocephala* groups (1.46 ± 0.67) (p < 0.001 and p < 0.01, respectively). At 6 h post-infection, *HSP70* expression in the 20% *L. leucocephala* group (2.59 ± 0.98) was significantly higher than that in the control group (1.00 ± 0.14) (p < 0.05). Markedly elevated expression was also observed at 12 h (53.11 ± 20.19) and 24 h (16.77 ± 6.19) (p < 0.001), as well as at 96 h (7.33 ± 5.00) and 120 h (6.97 ± 1.03) post-infection compared with the control group (p < 0.001).

In the 10% *L. leucocephala* group, *HSP70* expression tended to be higher than that in the control group, although significant differences were observed only at 6 h (4.36 ± 0.85) and 120 h (5.83 ± 1.73) post-infection (p < 0.001). Notably, at 6 h post-infection, *HSP70* expression was higher in the 10% *L. leucocephala* group than in the 20% *L. leucocephala* group.

*Cu/Zn-SOD* expression (Figure 6D) was generally higher in the 20% *L. leucocephala* group than in the control and 10% *L. leucocephala* groups throughout the experimental period. Significant upregulation in the 20% *L. leucocephala* group was observed at 0 h (7.02 ± 2.93) compared with the control group (1.07 ± 0.49) (p < 0.001) and the 10% *L. leucocephala* group (3.04 ± 0.30) (p < 0.01). At 6 h post-infection, *Cu/Zn-SOD* expression increased further to 15.32 ± 2.38 and remained significantly higher than in the control group (p < 0.001). Significant increases were also observed at 24 h (3.25 ± 1.33) compared with both the control and 10% *L. leucocephala* groups (p < 0.01 and p < 0.05, respectively), and at 48 h (3.79 ± 2.28) compared with the control group (1.06 ± 0.40) (p < 0.05).

In the 10% *L. leucocephala* group, *Cu/Zn-SOD* expression also tended to be higher than that in the control group, although the increase was less pronounced than that observed in the 20% *L. leucocephala* group. Significant differences between the 10% *L. leucocephala* and control groups were detected only at 6, 24, and 96 h post-infection (p < 0.01).

*ALF* expression (Figure 6E) was generally higher in the 10% and 20% *L. leucocephala* groups than in the control group. Significant upregulation was observed at 0, 12, 48, 72, and 120 h post-infection in both treatment groups compared with the control group, although no significant differences were detected between the 10% and 20% *L. leucocephala* groups at these time points. At 6 h post-infection, *ALF* expression in the 20% *L. leucocephala* group (18.44 ± 16.15) also showed an increasing trend; however, the difference was not statistically significant compared with the control group. In contrast, the 10% *L. leucocephala* group (23.01 ± 4.31) exhibited significantly higher *ALF* expression than the control group (1.89 ± 1.86) at this time point.

*C-lectin* expression (Figure 6F) at 0 h was significantly lower in the 10% *L. leucocephala* (0.16 ± 0.07) and 20% *L. leucocephala* groups (0.24 ± 0.17) than in the control group (1.04 ± 0.33). However, at 12 h and 48 h post-infection, *C-lectin* expression was markedly elevated in the 20% *L. leucocephala* group, reaching 52.56 ± 10.57 and 11.24 ± 3.54, respectively, which were significantly higher than those in the control group (1.03 ± 0.26 and 1.04 ± 0.37, respectively) and the 10% *L. leucocephala* group (7.60 ± 2.58 and 1.81 ± 1.58, respectively) (p < 0.001).

*C-lectin* expression in the 10% *L. leucocephala* group also showed an increasing trend during the 12–96 h post-infection period, similar to that observed in the 20% *L. leucocephala* group. At 72 h, expression in the 10% *L. leucocephala* group (30.26 ± 6.47) was significantly higher than that in the control group (1.01 ± 0.20) and the 20% *L. leucocephala* group (6.27 ± 2.26) (p < 0.001). After 72 h post-infection, *C-lectin* expression in the 20% *L. leucocephala* group gradually decreased, resulting in a significant difference compared with the 10% *L. leucocephala* group (p < 0.05).

### Mortality rates

**Figure 7:**
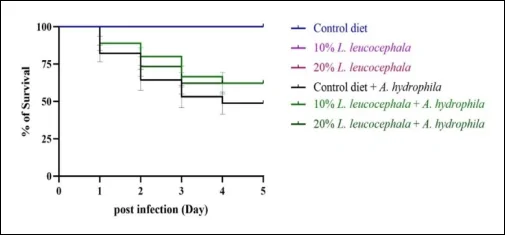
Survival rate of *Macrobrachium rosenbergii* following *Aeromonas hydrophila* infection. Data are expressed as mean ± SD (n = 3 replicate tanks per group). Survival analysis was performed using the log-rank (Mantel–Cox) test. No significant differences were observed between the control and 10% replacement groups (*p* = 0.167) or between the control and 20% replacement groups (*p* = 0.204).

The effect of 10% and 20% fermented *L. leucocephala* in fishmeal replacement on the survival of adult *M. rosenbergii* during *A. hydrophila* infection is shown in Figure 7. Survival of *M. rosenbergii* tended to be higher in the 10% and 20% *L. leucocephala* groups than in the control group; however, the differences were not statistically significant (p = 0.167 and p = 0.204, respectively). No dose-dependent effect was observed.

## DISCUSSION

### Functional value of fermented *L. leucocephala* as a fishmeal substitute

*L. leucocephala* leaves are widely recognized as a rich source of protein and essential minerals, making them a promising alternative feed ingredient for livestock and aquaculture species in tropical regions. Their high protein content is a key nutritional advantage, along with the presence of 17 flavonoids known for biological activities, including antioxidant and anti-inflammatory effects [14]. In the present study, *L. plantarum* TISTR1284 was used to improve the bioavailability and functional properties of *L. leucocephala* leaves. Fermentation is known to enhance food safety and nutritional quality by producing organic acids, including lactic, acetic, propionic, and formic acids, which help eliminate harmful bacteria and toxins while preserving sensory qualities [17]. However, the present experimental design does not allow separation of the individual contributions of fermentation, plant substitution, and probiotic effects; therefore, the outcomes observed here should be interpreted as the combined effect of the fermented ingredient rather than being attributed to a single factor.

*A. hydrophila*, a pathogenic Gram-negative bacterium, poses a serious threat to *Macrobrachium nipponense* and *M. rosenbergii* [34, 35]. Plant extracts from *Withania* *somnifera* and *Melaleuca cajuputi* have been shown to enhance immune responses and increase shrimp survival during *A. hydrophila* infection [36, 37]. *L. leucocephala* seed protein has also been shown to enhance bacterial disease resistance in the catfish *Clarias gariepinus* [12]. In the present study, dietary supplementation with fermented *L. leucocephala* leaves enhanced immune parameters and improved survival of *M. rosenbergii* following *A. hydrophila* challenge.

### Hemocyte proliferation and hematopoietic activity

Crustacean resistance to pathogens relies primarily on the innate immune system. Hemocytes are crucial cells that mediate physiological responses to infection and environmental stress. Probiotics and medicinal plant extracts have been applied to enhance immune parameters in *M. rosenbergii* [38, 39]. In the present study, increased circulating hemocytes and a higher number of mitotic cells in hematopoietic tissue were observed when fermented *L. leucocephala* leaves were added to the diet of *M. rosenbergii*. This response may contribute to accelerated maturation of hemocyte precursors in hematopoietic tissue, thereby maintaining hemocyte population and functionality during bacterial challenge [40].

*L. leucocephala* contains apigenin, kaempferol, and juglanin, which induce the transcriptional activity of pHRE-Luc before promoting erythropoietin expression and stimulating red blood cell production [14]. Upregulation of *CHF* is a biological indicator associated with hemocyte and hematopoietic cell proliferation in *M. rosenbergii* [27]. The increase in hematopoietic proliferation, reflected by *CHF* expression in hemocytes and hematopoietic tissue of the 10% and 20% *L. leucocephala* groups, suggests that fermented *L. leucocephala* promotes hematopoietic activity and hemocyte homeostasis during bacterial infection.

### PO activity and *proPO* expression

PO is an important enzyme that promotes humoral defense mechanisms and the elimination of pathogens in invertebrates [41]. Plant extracts have been shown to induce PO activity and improve immunocompetence against pathogens in crustaceans [38, 42, 43]. In the present study, *M. rosenbergii* fed fermented *L. leucocephala* showed increased PO activity in hemolymph and significant upregulation of *proPO*. Antimicrobial and antioxidative activities induced by phytochemicals in *L. leucocephala* have been previously reported [44]. These findings suggest that key bioactive properties of *L. leucocephala* were retained after fermentation, although the specific compounds responsible for the observed effects were not directly quantified in the present study.

### Modulation of Toll pathway-related signaling

*TRAF6* is a highly expressed gene that acts as a crucial signal transducer triggering downstream cascades mediated by TNF receptors and the IL-1 receptor/Toll-like receptor superfamily during immune responses [45]. In *Fenneropenaeus* *penicillatus*, higher *TRAF6* expression was correlated with peroxinectin expression after infection by white spot syndrome virus and *Vibrio alginolyticus* [46]. *TRAF6*-like transcription was upregulated after *Staphylococcus aureus* and *Edwardsiella* *ictaluri* challenge in *Procambarus clarkii* [47]. Knockdown of *TRAF6*-like inhibited downstream effector genes, including *Dorsal* [47]. *TRAF6* regulates immune responses during *A. hydrophila* infection because mannose-binding lectin and crustin are decreased after *TRAF6* silencing [30].

In the present study, *TRAF6* expression decreased within 48 h, and *Dorsal* expression declined at specific time points in the 10% and 20% groups. These findings indicate modulation of inflammatory signaling; however, bacterial burden or clearance was not quantified in this study. Therefore, improved survival should not be directly interpreted as increased bacterial clearance. Flavonoids in *L. leucocephala* have been reported to reduce pro-inflammatory cytokine secretion in RAW 264.7 macrophages [14], which may partly explain the moderated inflammatory profile observed here.

### IMD pathway and immune-related gene responses

**Two major pathways are involved in shrimp immunity:** the Toll pathway and the IMD pathway. These pathways share downstream signaling components, and crosstalk between them contributes to protection against bacterial infection [48]. In the present study, *C-lectin* was downregulated, whereas IMD-related genes (*IMD* and *Relish*) and effector/stress genes (*HSP70*, *Cu/Zn-SOD*, and *ALF*) were upregulated, particularly in the 20% group. This pattern suggests coordinated immune modulation rather than simple immune suppression. Such modulation may reflect regulated immune adjustment to dietary bioactive compounds, as previously observed in crustaceans exposed to functional feed additives and plant-derived extracts [49, 50].

However, the precise molecular mechanism underlying the activation of the immune pathway by fermented *L. leucocephala* remains unclear and warrants further mechanistic investigation. In particular, this study did not identify the specific bioactive compounds responsible for modulating the IMD pathway, although phytochemicals such as phenolics have been reported to influence immune responses in *C. gariepinus* [51]. Nevertheless, the exact molecular mediators and their direct interactions with IMD pathway components remain unclear and require targeted metabolomic and functional studies.

### Gut health, paraprobiotic effects, and postbiotic contributions

In addition to immunomodulation, *L. leucocephala* has been associated with gut health and nutrient utilization [52]. Replacing 25%–50% of fishmeal with *L. leucocephala* leaves in Nile tilapia diets did not adversely affect fish health, although some growth retardation was observed [11]. Improvements in digestibility and nutrient uptake have also been reported when *L. leucocephala* leaves are fermented with beneficial gut bacteria. For example, *Bacillus subtilis* and *Bacillus circulans* enhanced nutritional value and growth performance in *Labeo* *rohita* when included in diets containing 30%–40% *L. leucocephala* [53]. In crustaceans, host-associated probiotics such as *Lactococcus lactis* have shown similar benefits in *M. rosenbergii*, improving both growth and immune function [39].

Although viable LAB counts in the final pelleted feed and during storage were not quantified, the present findings suggest that the beneficial effects observed in *M. rosenbergii* were likely mediated not only by live probiotic cells but also by fermentation-derived paraprobiotics and postbiotics acting synergistically with enhanced plant bioactive compounds. Paraprobiotics, including inactivated microbial cells and cell-wall components such as peptidoglycans, teichoic acids, and surface proteins, are known to interact with crustacean pattern-recognition receptors, thereby stimulating hemocyte proliferation and activating downstream immune pathways, including the IMD–Relish signaling cascade observed in the present study. Concurrently, fermentation by *L. plantarum* may generate beneficial postbiotic metabolites, including organic acids, bacteriocins, and bioactive intracellular compounds, which may further contribute to immune modulation and disease resistance.

### Detoxification and antioxidant-related benefits

The present findings suggest that *L. plantarum* fermentation may help mitigate one of the major limitations associated with plant protein utilization, namely ANFs. As shown in Table 1, fermentation reduced mimosine levels by 42.18%, decreasing from 92.46 to 53.46 mg/100 g. Mimosine is a toxic non-protein amino acid known to impair nutrient utilization and physiological performance in animals. Furthermore, the incorporation of fermented *L. leucocephala* enhanced the phytochemical and antioxidant properties of the diets, as evidenced by increased TPC and TFC, higher FRAP values, and improved radical-scavenging activity, as indicated by lower DPPH and ABTS IC_50_ values in the 20% inclusion group. Collectively, these findings suggest that fermented diets may function as promising functional feeds enriched with detoxified plant matrixes, fermentation-derived metabolites, and microbial-derived bioactive components that collectively support innate immune responses in prawns.

### Study limitations and future research

One limitation of the present study is that the experimental diets were not strictly formulated to be isonitrogenous. As shown in Table 2, crude protein content decreased from 44.63% in the control diet to 36.75% in the 20% replacement group, representing a substantial nutritional variation that may have influenced the physiological and immunological responses of *M. rosenbergii*. Therefore, the effects cannot be attributed exclusively to the bioactive properties of fermented *L. leucocephala*, as differences in dietary protein levels may also have contributed to the observed responses. In addition, crystalline amino acids such as lysine and methionine were not supplemented to achieve precise amino acid balancing among the experimental diets. Consequently, variations in essential amino acid composition resulting from the inclusion of fermented *L. leucocephala* may have further influenced immune and physiological responses. Future studies using strictly isonitrogenous, isolipidic, and amino acid-balanced diets are required to better distinguish the specific functional effects of fermented *L. leucocephala* from potential nutritional confounding factors.

Another limitation is that the feeding trial duration was relatively short compared with conventional aquaculture nutrition studies, and growth performance parameters such as weight gain, specific growth rate, and feed conversion ratio were not evaluated. In addition, pellet water stability and nutrient leaching rates were not quantitatively assessed. Since feed stability in water may influence nutrient availability, feed intake, and feeding efficiency in prawns, these parameters should be evaluated in future studies to better characterize the physicochemical quality and practical applicability of the experimental diets under aquaculture conditions. Therefore, the long-term nutritional suitability and commercial applicability of the diets could not be fully determined in the present study. Furthermore, the current experimental design does not allow precise determination of the optimal inclusion level or dose-dependent immune responses associated with fermented *L. leucocephala* supplementation.

The present study also has limitations related to fermentation characterization and microbial analysis. Post-fermentation LAB viability, pH, lactic acid concentration, titratable acidity, and detailed biochemical characteristics of the fermented product were not directly quantified. In addition, the microbial composition of the fermented material and viability of *L. plantarum* in the final feed and during storage were not evaluated. Consequently, microbial dynamics and fermentation-derived metabolites associated with the fermented product could not be fully characterized. Therefore, the relative contributions of live probiotic cells, postbiotic metabolites, and paraprobiotic effects remain unclear. Moreover, microbial safety analyses, including assessments of pathogen and mycotoxin contamination, were not performed directly, although fermentation was conducted under sealed, anaerobic conditions to minimize contamination risk. Future studies integrating comprehensive fermentation profiling, microbial safety analyses, metabolite characterization, and gut microbiota analysis would provide stronger mechanistic insight into host–microbe interactions and the functional properties of fermented *L. leucocephala*.

Antioxidant capacity was not directly measured in hemolymph or tissues of *M. rosenbergii*. The present study primarily evaluated antioxidative responses through immune- and oxidative stress-related gene expression, particularly *Cu/Zn-SOD* expression in hemocytes. Therefore, the antioxidative effects of fermented *L. leucocephala* supplementation should be interpreted as indirect molecular evidence rather than direct biochemical confirmation of antioxidant activity.

Several limitations were also associated with the bacterial challenge experiment. Unchallenged control prawns were not sampled at all post-challenge time points (6–120 h), limiting the ability to distinguish the direct effects of dietary supplementation from diet × infection interaction effects. In addition, bacterial load in hemolymph or tissues was not quantified. Therefore, the improved survival and immune responses observed in the treatment groups cannot be directly interpreted as evidence of enhanced bacterial clearance capacity. Correlation analyses between immune parameters and survival outcomes were also not performed, preventing detailed evaluation of the quantitative relationship between immune modulation and disease resistance. Future studies incorporating time-matched unchallenged controls, quantification of bacterial load, and integrated multivariate statistical analyses would strengthen the mechanistic interpretation of immune responses and disease resistance in *M. rosenbergii*.

Despite these limitations, the consistent enhancement of several immune parameters and improved resistance to *A. hydrophila* observed in the treatment groups suggest that fermented *L. leucocephala* may contribute beneficial immunomodulatory effects in prawns. Importantly, no adverse effects on intestinal morphology were detected, supporting the safety of the dietary inclusion levels used in the present study. Nevertheless, the findings should be interpreted with caution, and further long-term studies using rigorously controlled diet formulations and comprehensive physiological, microbiological, and biochemical evaluations are warranted to clarify the specific contribution of fermented *L. leucocephala* to immune modulation and disease resistance in prawn aquaculture.

## CONCLUSION

Dietary supplementation with fermented *L. leucocephala* enhanced several innate immune responses in *M. rosenbergii* challenged with *A. hydrophila*. Fermentation improved the nutritional quality of *L. leucocephala* by increasing essential amino acid content and reducing mimosine concentration, while supplementation of experimental diets increased TPC, TFC, antioxidant activity, and reducing power. Prawns receiving fermented *L. leucocephala*, particularly at the 20% inclusion level, exhibited enhanced hemocyte proliferation, increased hematopoietic activity, elevated PO activity, and upregulated expression of key immune- and stress-related genes, including *CHF, proPO, IMD, Relish, HSP70, Cu/Zn-SOD,* and *ALF*. These responses were accompanied by improved resistance to *A. hydrophila* infection and the maintenance of normal intestinal morphology, indicating that the evaluated dietary inclusion levels were well tolerated.

From a practical perspective, the findings suggest that fermented *L. leucocephala* has potential as a functional feed ingredient and partial fishmeal substitute for freshwater prawn culture, providing both nutritional and immunological benefits. The study further demonstrates that fermentation with *L. plantarum* may reduce ANFs while enhancing the bioactive and antioxidant properties of plant-derived feed ingredients.

A major strength of this study is the comprehensive evaluation of immune responses through hematological, histological, molecular, and antioxidant-related assessments following bacterial challenge.

Future studies should employ isonitrogenous and amino acid-balanced diets, evaluate long-term growth and production performance, characterize fermentation-derived metabolites and microbial dynamics, quantify pathogen clearance, and investigate gut microbiota responses. Such studies will help clarify the mechanisms underlying immune modulation and establish the optimal inclusion level of fermented *L. leucocephala* for commercial aquaculture applications.

Overall, the present study provides evidence that fermented *L. leucocephala* can serve as a promising functional feed ingredient that enhances innate immunity, antioxidant status, and disease resistance in *M. rosenbergii*, supporting its potential application in sustainable prawn aquaculture.

## DATA AVAILABILITY

The supplementary data can be made available from the corresponding author upon request.

## GENERATIVE AI DECLARATION

The authors declare that generative artificial intelligence (AI) tools were used solely to improve language, grammar, and readability during manuscript preparation. All scientific content, data analysis, interpretation of results, and conclusions were developed and verified by the authors. The authors take full responsibility for the accuracy, integrity, and originality of the work presented, and no AI tool was listed as an author.

## AUTHORS’ CONTRIBUTIONS

AP: Methodology, investigation, validation, formal analysis, data curation, visualization, and manuscript drafting. LP and KJ: Methodology, investigation, and data curation. SlS, SkS, PaP, and PoP: Bacterial culture and feed pellet preparation. SuS: Formal analysis and data curation. RV and CC: Validation, formal analysis, visualization, conceptualization, manuscript review, and editing. All authors have read and approved the final manuscript.
